# The Treatment of Hemorrhoids by Ligature, by Injection, and by the Clamp and Cautery

**Published:** 1898-04

**Authors:** Herman E. Pearse

**Affiliations:** Professor of Anatomy, Kansas City Medical College; Consultant to the Kansas City, Fort Scott & Memphis R. R. Hospital; Editor “The Kansas City Medical Index,” etc.


### Abstracts and Selections.

### The Treatment of Hemorrhoids by Ligature, by Injection, and by the Clamp and Cautery.

BY HERMAN E. PEARSE, M. D.,
Professor of Anatomy, Kansas City Medical College; Consultant to the Kansas City, Fort Scott & Memphis R. R. Hospital;
Editor "The Kansas City Medical Index," etc.

[Read before the Central District Medical Society, at Sedalia, Mo., November 4, 1897. From Kansas Medical Journal, November 20, 1897.]

The victim of hemorrhoids sooner or later comes for relief to his doctor. He will ask first a prescription; that failing, a treatment of some kind; last of all an operation under chloroform.

The anatomy of the parts explains well the classification of external and internal hemorrhoids. The former are derived from the inferior and middle hemorrhoidal veins, and the latter from the superior hemorrhoidal. The superior hemorrhoidal veins supply the rectum above the sphincters—have no valves, and empty into the mesenteric vessels and thence into the portal circulation. These form the internal pile (where it is a venous tumor, as it generally is in the beginning), and is caused by any obstruction to the circulation through, or disease of, the visceral venous system, as in liver diseases, retroversion of the uterus, stricture, etc. The middle and inferior hemorrhoidal plexuses supply the anus and contiguous structures, and empty into the iliac veins and thus into the right heart via the vena cava. Their dilatation becomes a fact when disease of the general venous system is at fault; as in protracted alcoholism, hard coughing, lifting, etc.

The two anastomose, thus forming one of the means of collateral circulation when cirrhosis or other obstructive disease cuts off the portal circulation by pressure.

Clinically, however, we speak of external and internal piles according as they are above or below the grip of the sphincter, and we call them capillary, arterial or venous as they show a preponderance of these blood vessels.

As to treatment (the subject of this paper), we have three principal lines of attack, the injection of caustic and astringent substances, the ligature and excision, and the clamp and cautery. The injection method can be used on all kinds of cases—external, capillary, venous, strangulated and ulcerated, yet it is not desirable so to use it; in fact, the real usefulness of the method rests in the treatment of internal hemorrhoids of limited number and small size, that have not much ulceration; yet, I repeat, that with a good fluid and due care in its use it is possible to cure almost any case of hemorrhoids by injection, if the patient will spend time enough for the treatment to be completed. When inflamed and engorged, it is better if the parts be relieved by a few days of preparatory treatment, which should consist of hot douches, rest, and cathartic doses of sulphur and cream of tartar to unload the portal circulation. When ready for treatment the patiennt is placed on his side, with limbs drawn up and the tumors brought into view. A five per cent. solution of cocaine applied to the parts, relieves the pain, and a little straining will cause the pile tumors to protrude.

The tumor selected is then punctured with a fine hypodermic needle, attached to an ordinary hypodermic syringe, and a few drops of the fluid deposited into its tissue. In pendant tumors get into the center. In sessile tumors inject into their bases. Be careful not to inject the fluid into the muscular tissue of the bowel, as it will cause sloughing of tissue and prolonged suffering. The portion of the tumor that has been sufficiently injected will turn a dull grayish color, showing the death of the tissue. Where in doubt as to the amount to inject, use two or three drops, and hold the needle in situ while awaiting its action. A few more may be required. I have used the following fluid with success, the formula for which I have taken from Dr. Agnew, of San Francisco, who published it in his book on this treatment.

SOLUTION 1.

℞ Plumbi acetatis,

Sodii biboratis ..........................aa ℨ ij

Price's glycerine............................℥ i

Mix in a graduate and let stand in a warm water bath for twenty-four hours.

SOLUTION 2.

℞ Calvert's No. 1 carbolic acid cryst......℥ j

Aqua destil.......................... ........ℨ ij

When well dissolved, add six drams of No. 1 to ten drams of No. 2.

This makes a solution that turns pink on standing, and, unless warm, is turbid. It should be warmed before using. Only one, or at most two tumors, should be treated at one sitting. If there is much pain, hot sitz baths, opium and belladonna, or atropine, should be given. The anus should be covered with a pad of soft cotton or gauze smeared with vaseline. The bowels should be kept loose by sulphur in some form, either with cream of tartar or in the compound licorice powder. Wyeth & Bros. make a very elegant tablet containing five grains each of sulphur and cream of tartar, which is a desirable form for administration.

CASE I.—Mr. S——, of Kansas City, Mo., laborer, employed in lifting and loading freight into cars at the freight depot, came to me February 21. His piles were four in number, ulcerated, inflamed, bleeding, and so sore that he could not work. He absolutely refused operation under anesthesia, and had tried many forms of ointments. I asked for two months to cure him, which he accepted. I kept him in bed one week, applying hot fomentations, when he came to my office for treatment.

After brushing the parts with cocaine, and placing a hot, moist towel against them, they came down readily, with a little straining, bleeding freely. I selected the worst looking one, and after five minutes spent in slowly injecting about twelve drops of the solution, had the pleasure and satisfaction of seeing it turn a dull gray color aud shrink down. He returned every five days, and was injected four or five times at intervals of five to ten days. He resumed work after the first injection, keeping the parts covered into the following:

℞ Fl. ext. hamamelis............................ʒ i

Powdered opium............................ʒ ss

Per-sulph. iron..................................ʒ i

Vaseline..............................................℥ j

Without further incident he continued his work and treatment, and was discharged perfectly cured. He is paying his bill by monthly installments, and each month expresses his delight at his easy cure. Should a method so useful be avoided because, forsooth, the ignorant quack has used it?

The clamp and cautery is a valuable method where the growths can be removed at one sitting under anesthesia. I wish to say here that I much prefer to operate under chloroform upon these cases, for the following reasons:

1. It is more pleasant for the operator, and he can do better work under general than under local anesthesia.

2. The sphincter can be widely dilated, which alone is sometimes sufficient to cure the case, and always aids materially in cure by any method.

3. It is all done at one sitting, and the cure is thus more rapid and satisfactory.

I present the instruments used in the operation. The cautery is a Paquelin, and is at present charged with gasoline from the grocery. It works all right. The clamps are the pattern of Smith, Kelsey and Gant. I believe the Gant clamp is best. I like it best. I have operated with only a tissue forcep for a clamp, and succeeded, but it is not powerful enough to crush the tissue into firm pedicle. It is best to divide the skin and mucous membrane at the base, fit in the clamp, close it, cut off the tumor with curved scissors, and roast the stump with the cautery at red heat. There is need to see that not only is the cut edge seared, but that the stump is hardened by the heat. The after cure and dressing is the same as in case of injection. The patients go about in three or four days, and are entirely well in as many weeks, often in much less time.

The operation by ligature is the next to be considered, and is adapted, like the foregoing, to all severe cases of internal piles where excision under chloroform is allowed. The patient's bowels are emptied the previous day and cleansed by enema a few hours before operating. When anesthetized he is placed in lithotomy position, the sphincter well dilated and the skin and mucous membrane divided, a curved needle threaded with strong silk is passed through the center of the base, the needle cut off, and the two remaining ligatures tied both ways about the tumor, sinking the thread into the line of incision.

Case II.—*Clamp and Cautery.*—Mr. G— —, farmer, had old hemorrhoids for six years. Was in bed with an attack of the inflammation of the tumors, and had been for a week. Under his doctor's care he was treated a few days with hot fomentations and laxatives. The soreness, however, did not disappear. He accepted offer of operation, and was anesthetized August 16th. Three stumps were made, each in succession being brought down and the base encircled by an incision running through the mucous membrane and skin. To each in turn the clamp (Kelsey's) was applied, and the tumor cut away with curved scissors. An assistant meanwhile was preparing the cautery, and the stump was immediately and thoroughly seared. The patient had retention of the urine, and suffered some pain the first thirty-six hours, but the parts were kept smeared with vaseline containing belladonna and carbolic acid and one-fourth grain morphine administered hypodermically. On the 20th he was in the yard, and on the 22d, seven days after operating, rode to town in his wagon. The bowels were kept loose with compound licorice powder at bed time. His recovery was perfect.

Case III.—Mr. G———, of Stockton, Mo., had been a severe sufferer from piles for about three years. As often happens, much of the pain was caused by a fissure, though attributed to the pile tumors. Operation was accepted and performed August 21. The bowel was prepared by his doctor, Dr. E. A. Smith, the day before, and an enema given an hour and a half before the operation. Under anesthesia the anus was thoroughly dilated and the tumors brought down. Each was in turn grasped by the pipe forcep, and the base encircled with a cut running through the skin and mucous membrane. The curved needle was then passed through the base of the tumor and a double silk ligature drawn through, the needle cut off, and the two ligatures thus formed carried, one forward, and the other backward, and securely tied, sinking them well into the cut. The tumor was then cut off. The fissure was curetted, the sphincter again stretched and the rectum, which was much ulcerated and bleeding, packed with iodoform gauze. The packing was removed in thirty-six hours, and the parts smeared with carbolized vasilene and a pad of gauze placed over it. Two ligatures came away the fifth day, and the third on the seventh day. Three weeks later the doctor wrote that the man was well and could walk, work, and sit down without pain, and bowel movements were free and painless.

DANGERS.

1. *Injection Method.*—Extensive slough, and pain from inflammation of cellular tissue beyond the pile.

2. *Ligature.*—Retention of urine; swelling of parts with pain; tetanus.

3. *Clamp and Cautery.*—Retention of urine; pain and swelling; free hemorrhage in twelve to twenty hours; tetanus.

4. *All.*—Shock from operation; secondary hemorrhage.

I have never seen serious trouble or death follow an operation for hemorrhoids.—*Kansas City Medical Index.*

